# Protocol for a scoping review to map patient engagement in scoping reviews

**DOI:** 10.1186/s40900-022-00361-x

**Published:** 2022-06-20

**Authors:** Nebojša Oravec, Caroline Monnin, April Gregora, Brian Bjorklund, Mudra G. Dave, Annette S. H. Schultz, Anna M. Chudyk

**Affiliations:** 1 Max Rady College of Medicine, Rady Faculty of Health Sciences, University of Manitoba, AE101 - 820 Sherbrook Street, Winnipeg, MB R3A 1R9 Canada; 2 Neil John Maclean Health Sciences Library, University of Manitoba, 727 McDermot Avenue, Winnipeg, MB R3E 3P5 Canada; 3 Enhanced Recovery Protocols for Cardiac Surgery Patient Researcher Group, St. Boniface Hospital, 369 Taché Avenue, Winnipeg, MB R2H 2A6 Canada; 4 Cardiac Sciences Program, CR 1005 - St. Boniface Hospital, 369 Taché Avenue, Winnipeg, MB R2H 2A6 Canada; 5 College of Nursing, Rady Faculty of Health Sciences, University of Manitoba, 89 Curry Place, Winnipeg, MB R3T 2N2 Canada; 6 Health Services and Structural Determinants of Health Research, St. Boniface Research Centre, 351 Taché Avenue, Winnipeg, MB R2H 2A6 Canada

**Keywords:** Patient and public involvement, Patient-oriented research, Knowledge synthesis, Review, Health research, Patient consultation

## Abstract

**Background:**

Scoping reviews of health research are increasing in popularity. However, only a minority of scoping reviews in this sector engage patients and caregivers as co-producers of the research. Despite developments in scoping review methodology, which insist that stakeholder consultation is essential, no guiding methods exist to instruct the conduct of this stage. Thus, it is necessary to understand how patients and caregivers have been engaged as part of scoping reviews, toward a unifying methodology.

**Methods:**

We have developed a protocol for a scoping review of methods used to engage patients and caregivers in scoping reviews of health research. The search strategy will comprise two phases: the first will involve a secondary analysis of retrieved articles from a prior scoping review, and the second will identify articles that cite Levac et al.’s update to the original scoping review framework by Arksey and O’Malley. Titles and full texts of retrieved articles will be screened in duplicate. Inclusion will be limited to articles related to heath research that follow the six-stage scoping review framework by Arksey and O’Malley and that report patient engagement activities during at least one stage. The method of analysis of charted variables will be decided once data have been collected. Two patients will be engaged as collaborators throughout this review. We will also consult with patients, caregivers, and researchers upon completion of preliminary analyses.

**Discussion:**

We anticipate that our scoping review will provide guidance for researchers seeking to involve health care stakeholders as co-producers of scoping reviews.

**Supplementary Information:**

The online version contains supplementary material available at 10.1186/s40900-022-00361-x.

## Plain English summary

A “scoping review” is a type of study that collects and summarizes published and unpublished research reports to better understand the amount and types of information available on a particular topic. There is a well-known framework for how to conduct a scoping review, which involves six stages. The sixth stage is optional, and involves consulting with people who have an interest in the research results (i.e., people who the research is “about” or who it will affect the most). Very few scoping reviews actually include this stage, potentially due to a lack of practical guidance on how to perform it. For scoping reviews related to health research, it is important to consult or more widely engage patients and caregivers in the scoping review’s conduct because these individuals have a unique type of knowledge that comes from their experience of a health issue, which can yield valuable insights. Therefore, we have designed a scoping review that will identify the ways in which patients and caregivers have been engaged in scoping reviews of health research in the past. We hope to produce recommendations to make it easier for other researchers to engage patients and caregivers in scoping reviews.

**Supplementary Information:**

The online version contains supplementary material available at 10.1186/s40900-022-00361-x.

## Background

A scoping review is a type of knowledge synthesis that aims to map the key concepts and knowledge gaps related to an exploratory research question, and the sources and types of evidence available [1–3]. Its popularity has skyrocketed over recent years [4]. Several approaches have been proposed for conducting scoping reviews [5–8], of which the framework by Arksey and O’Malley is one of the most common [8]. According to Arksey and O’Malley, a scoping review consists of six stages: (1) Identifying the research question, (2) Identifying relevant studies, (3) Study selection, (4) Charting (i.e., extracting) the data, (5) Collating, summarising, and reporting the results, and (6) Consultation. Of these stages, the sixth (consultation) was considered optional. Though Arksey and O’Malley discuss consultation as providing an additional source of knowledge, particularly on the relevance of data gathered from studies included in the scoping review, they do not offer advice on how to define or conduct the consultation, collect and organize the resultant information, nor how to integrate the results of consultation into the findings of the scoping review. Other barriers to consultation could be related to resources, skills and motivations, recruitment issues, and logistics [9]. Unsurprisingly, less than 50 percent of scoping reviews incorporate consultation within their methods [8].

Consultation has been conceptualized as an activity in which relevant stakeholders provide feedback to researchers, who may or may not incorporate this information in their subsequent decision-making [10, 11]. Patient engagement is a research approach that involves the active engagement of patients and informal caregivers (e.g., friends and family) in the design, conduct, analysis, and/or dissemination of research—also referred to as “co-production” [12]. In this context, consultation is one mode along a “spectrum of engagement,” where each level across the spectrum is associated with a different degree of decision-making power among researchers and the public [10]. For example, patients have an advisory role in the level “involve,” whereas there is equal partnering in the level “collaborate,” and they have final decision-making power in the level “lead/support.” The moral imperative of engaging patients and caregivers in research through activities such as consultation, and its benefits for public funders, participants, healthcare users, academia, and industry, is becoming increasingly apparent [13, 14]. Some of these benefits include the potential for increased uptake of research findings, results that are more generalizable, and increased satisfaction. In an update of the framework proposed by Arksey and O’Malley, Levac et al. argue that consultation should be a requirement [6]. Thus, failure to engage patients and caregivers in scoping reviews can be seen as a limitation, especially in the realm of health and medicine, which is the sector that publishes the majority of scoping reviews [8].

To support the uptake of consultation as a required component of scoping reviews, in this article we propose a protocol for a scoping review of methods to engage patients and caregivers in scoping reviews of health research. Since we conceptualize consultation as one component of a broader engagement spectrum that represents the different degrees to which patients and caregivers can be engaged in scoping reviews, we are not only interested in studies that “*consult*” patients and caregivers but also in the broader spectrum of engagement modes (i.e., involve, collaborate, lead/support). Further, as the spectrum of engagement provides limited practical guidance on the types of activities that can be used to engage patients and caregivers, we are interested in not only the levels at which patients and caregivers were engaged, but also the activities used to engage them. Through this proposed work, we hope to provide guidance on how patients and caregivers can be involved throughout the stages of a scoping review and thereby help facilitate the routine engagement of the stakeholders in this work. The results of the review will serve to emphasize consultation as a feature that distinguishes scoping reviews from other types of secondary research.

## Methods

A scoping review approach was chosen due to the broad nature of our research question. Moreover, we anticipate heterogeneous methods for and ways of reporting patient engagement, primarily because the topic of engagement methods in context of health-related scoping reviews has not been extensively studied before.

### Team composition and engagement of patients within our research team

Uniquely, our entire research team (including two patient co-researchers, also commonly referred to as "patient partners" or "research collaborators") has experience engaging patients as co-producers of prior scoping reviews. The two patient co-researchers (AG and BB) have lived experience as cardiac surgery patients, previous co-research experiences, and have co-authored other publications and knowledge translation products [15, 16]. They collaborated on the development of this protocol through research team meetings and provided feedback on this resultant protocol submission. As we move forward on the scoping review, these patient co-researchers will continue to collaborate on the study as equal members of our research team. In this way, the study will serve as a working example of the benefits of co-production. We will formally establish their roles and responsibilities (along with those of the other research team members working on the study) through the co-development of a terms of reference at the outset of the scoping review. This living document will briefly explain all of the proposed stages of the scoping review, opening the door for informed conversations about the different opportunities for involvement across the entirety of the study. Based on early discussions, we anticipate that some of the patient co-researchers’ future roles and responsibilities may include engaging in team meetings aimed at: discussing challenges and uncertainties related to study selection and refining the search strategy and selection criteria if necessary, calibrating the study’s charting form based on a subset of extracted studies, deciding upon the best methods to analyze the charted variables, developing the instrument used in the study’s consultation phase, interpreting the collected data, helping plan the resultant manuscript and other related knowledge translation/dissemination products. In addition, patient co-researchers will provide feedback on the resultant scoping review manuscript and likely help create and present other knowledge translation/disseminations products. We will evaluate our study’s patient engagement activities and their impacts through a modified version of the Public and Patient Engagement Evaluation tool and regular reflexive check-ins focused on addressing the considerations related outlined in the Guidance for Reporting Involvement of Patients and the Public 2 (GRIPP2) checklist [17, 18].

### Reporting guidelines

The planning of this scoping review protocol follows the Preferred Reporting Items for Systematic Review and Meta-Analysis Protocols (PRISMA-P, Additional file 1) and the PRISMA-ScR (Additional file 2) [19]. The reporting of patient engagement activities is in accordance with the GRIPP2 short form (Additional file 3) [18]. There has been no prospective registration in any database.

### Scoping review methodology

This protocol describes a scoping review in keeping with the six-stage scoping review framework described by Arksey and O’Malley and with the revised recommendations by Levac et al. [5, 6].

### Stage 1: Identifying the research question

The research question was developed in accordance with the population, context, concept (PCC) framework (Table 1).

**Table 1:** Population, concept, context (PCC) framework for identifying the main concepts within the research question

Criteria	Determinants
**Population**	**Health research** The process for systematic collection, description, analysis, and interpretation of data that can be used to improve health [20]. Health research may include biomedical, clinical, health systems and services, and social, cultural, environmental, and population health studies [21].
**Concept**	**Methods to engage patients and caregivers in scoping reviews** *Engagement* is conceptualized as existing along a spectrum where the roles of patient and caregiver co-researchers vary according to the directions in which information flows and who holds decision-making power between researchers and patients and caregivers [10]. *Patient* is an overarching term that is inclusive of individuals with personal experience of a health issue or accessing the healthcare system, and informal caregivers, including family and friends [12]. *Caregiver* refers to those persons with interest in the patient’s health and wellbeing who are not remunerated for their role in the patient’s life [15].
**Context**	**Arksey and O’Malley six-stage scoping review framework** This framework proposes a sixth (historically optional) stakeholder consultation stage. As we conceptualize consultation as one component of a broader engagement spectrum [10] that represents the different degrees to which patients and caregivers can be engaged in scoping reviews, we are not only interested in studies that “*consult*” patients and caregivers, but also in the wider spectrum of engagement modes (i.e., consult, involve, collaborate, lead/support). *Stakeholder* is defined as a person who has an interest in the results of a research topic. In our study, stakeholders are patients and caregivers who have been engaged in scoping reviews of health research.

#### The research question

How have patients and caregivers been engaged in scoping reviews of health research guided by the methodology proposed by Arksey and O’Malley [5] and Levac et al. [6]?

### Stage 2: Identifying relevant studies

#### Search methods

The search strategy will comprise two phases that will be conducted and finished within approximately two weeks. Since Pham et al. conducted a comprehensive scoping review that identified all relevant scoping reviews published before 2013, our first phase will comprise a secondary analysis of the articles included in Pham et al.’s scoping review [8]. The authors’ search strategy identified all scoping reviews published in the following electronic databases within the associated timelines: Medline/PubMed (biomedical sciences, 1946–2012), SciVerse Scopus (multidisciplinary; 1823–2012), CINAHL/EBSCO (nursing and allied health; 1981–2012), Current Contents Connect/ISI Web of Knowledge (multidisciplinary current awareness; 1998–2012). Their strategy was also supplemented by a grey literature search and reference checking.

In the second phase, articles published subsequent to the last search date reported in Pham et al.’s review (October 1, 2012) will be identified through a search strategy designed to identify any scoping reviews citing Levac et al.’s 2010 update to the Arskey and O’Malley framework [6]. Scoping reviews citing Levac et al. will be identified from the following electronic databases: Medline (Ovid), Scopus, Web of Science, PsychINFO (Ovid), and the Joanna Briggs Institute. These databases were selected because of their relevancy to health research as well as their ability to track citations. Although Google Scholar can track citations, it was not selected due to its vast subject coverage. To locate grey literature, we will search ProQuest Theses & Dissertations and OSF Registries, using a similar search strategy of locating records that cite Levac et al. A sample search strategy is provided in Table 2.

**Table 2:** Sample search strategy (Phase 2)

Ovid MEDLINE(R) and Epub Ahead of Print, In-Process, In-Data-Review & Other Non-Indexed Citations and Daily <1946 to January 14, 2022> -------------------------------------------------------------------------------- 1 "20854677".rz. [PMID of Levac et al. 2010 article] (1864) 2 levac.ab,kw,kf. (225) 3 "scoping studies advancing the methodology*".af. (3) 4 1 or 2 or 3 (1929) 5 limit 4 to ed = 20110618–20220101 [Date of publication of Levac et al. 2010 article to present](1439)	

This two-phase search strategy was developed in an iterative process led by an expert health sciences librarian (CM) and validated by a second librarian using the Peer Review of Electronic Search Strategy (PRESS) checklist [22]. To maximize available resources, we focused our search strategy on scoping reviews that cite Levac et al.’s work, as they are often cited as supporting consultation as a required stage within Arksey and O’Malley’s framework. While we acknowledge that this decision could result in the omission of some relevant articles, through this approach, we aimed to balance comprehensiveness and feasibility in the context of our broad research question and a substantial increase in the number of scoping reviews published since the article by Pham et al. That is, although ideally we would wish to replicate the search conducted in Pham et al. with updated timelines, the growth of scoping reviews in the last 10 years makes it unfeasible to do so. For example, the Medline (Ovid) search conducted by Pham et al. in 2011 had 454 results, whereas the same search in Medline (Ovid) as of January 1, 2022 has 10,389 results. In addition, through hand-searching the literature, we have found that consultation methods are rarely mentioned in titles and abstracts, making it difficult to identify articles involving patient engagement using keyword search terms.

All resources will be exported to EndNote (version × 9), and the screening process will be conducted in the free, online systematic review management software, Covidence.

#### Inclusion and exclusion criteria

To be eligible for inclusion, a scoping review must be related to health research. Secondly, it must be guided by the Arksey and O’Malley framework for scoping reviews [5]. Even if not explicitly stated, if the scoping review comprises the same stages as those proposed by Arksey and O’Malley, it will be included. Finally, the scoping review must have engaged patients and/or caregivers (see Table 1 for definitions of these terms) in at least one of the stages of the scoping review, including and not limited to the formal consultation stage proposed by Arksey and O’Malley (i.e., Stage 6). Articles written in a language other than English will be excluded.

### Stage 3: Study selection

**Fig. 1:**
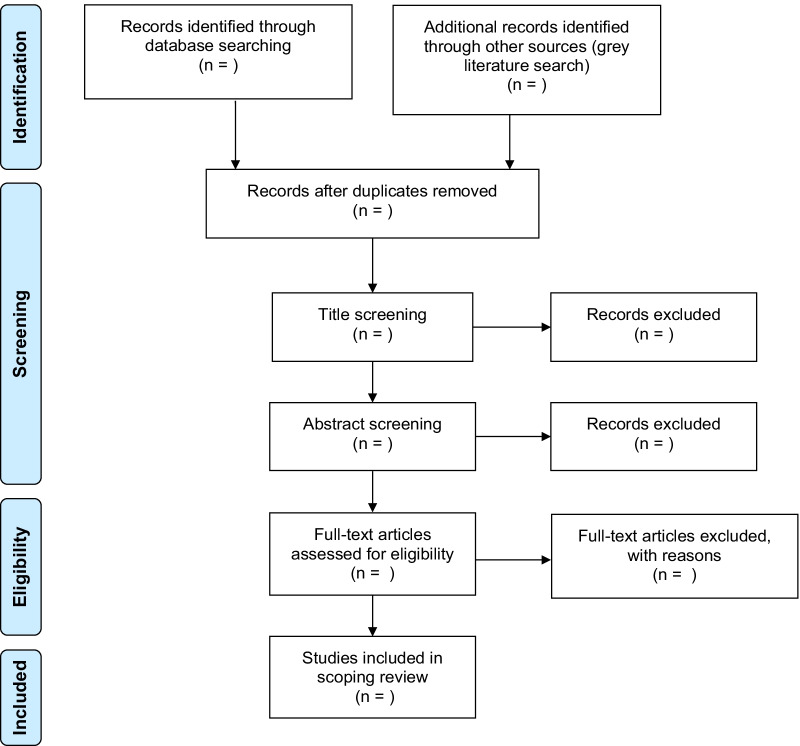
PRISMA flowchart for study selection

We will select articles using a two-phase approach: first from the list of included articles in the paper by Pham et al., then from the articles identified through our search. For both phases, the (1) titles and abstracts and (2) full-texts of identified articles will be screened in duplicate by two independent reviewers. This is instead of the standard three-step workflow (i.e., titles, then abstracts, then full-texts) for study selection, which is not relevant for our purposes because some of our inclusion criteria are not consistently reported in abstracts (i.e., scoping review framework and the presence of patient engagement).To establish agreement and resolve discrepancies in applying study selection criteria, reviewers will screen a random subset of titles and abstracts (n = 100 articles) and full-texts (n = 5 articles) and meet with a third reviewer to go over their agreement and resolve any discrepancies. Further, at the beginning and end, and throughout the study selection stage, reviewers will meet with the study team to discuss challenges and uncertainties and refine the search strategy or selection criteria if necessary. A third reviewer will resolve discrepancies as necessary. In the event that it is unclear whether a study meets inclusion criteria, a designated reviewer will attempt to contact the study’s corresponding author for clarifying information. The flow of studies included in the scoping review will be reported in a modified PRISMA flowchart (Fig. 1).

### Stage 4: Charting (i.e., extracting) the data

The charting form will include general information about the study and its design and more specific information about the methods of engagement (Table 3). Many of the engagement variables will be charted narratively (as opposed to nominal response choices) since there is no consensus on what elements of consultation (and more broadly, engagement) should be reported in a scoping review. The charting form will be calibrated by the research team using the first 5–10 studies to ensure consistency and may be modified in iterations based on increased familiarity with the included studies. Data will be charted in duplicate, and records will be stored and managed using Google Forms, Google Sheets, and Microsoft Excel. Inconsistencies will be resolved through conversation and/or consulting a third reviewer. To establish agreement and resolve discrepancies in how data are charted, at this stage’s outset, reviewers will chart a random subset (n = 5) of included studies and meet with a third reviewer to go over their agreement and resolve any discrepancies. To help minimize missing data, a designated reviewer will attempt to contact the corresponding author of any included study that is missing information relevant to the charting form.

**Table 3:** Proposed charting form

Category	Variable
**Publication characteristics**	Title
	Year of publication
	Journal (or source if unpublished)
	Published (yes/no)
**Author characteristics**	Surnames
	Corresponding author contact information
	Countries
	Discipline; point of view; “lens”
**Study characteristics**	Health topic
	Design
	Primary outcome(s)
	Outcome measures
	Type of health research (e.g., basic science, clinical, epidemiology)
	Limitations/risk of bias
**Stakeholder (i.e., patient or caregiver) characteristics**	Who was engaged in the scoping review (patients, caregivers, both, other)?
	Total number of stakeholders engaged
	What perspectives did the stakeholders represent?
	Average stakeholder age
	Stakeholder ethnicity/race
	Stakeholder gender
	Describe any other information provided about the stakeholders that was unrelated to their identity as a patient or caregiver.
	During which Arksey and O’Malley scoping review stages were stakeholders engaged (i.e., 1–6, N/A, unclear)?
	How were they stakeholders engaged (method of engagement)?
**Consultation/engagement characteristics**	What level along Manafo et al.’s [10] revised engagement spectrum best describes the engagement approach taken and why? Please indicate whether this was your perception vs. stated by the study authors.
	What was the established purpose of engaging stakeholders?
	How did researchers keep track of stakeholder contributions (e.g., notes)?
	How were stakeholder contributions incorporated and/or analyzed (e.g., thematic analysis, revision of preliminary framework)?
	How did stakeholder contributions influence/impact the research and how was this measured?
	Describe any strengths and limitations mentioned relevant to the engagement approach or process
	What challenges to engagement were reported (if any)?
	Which patient engagement reporting guideline was used, if any (e.g., GRIPP-2)?
	Were stakeholders compensated (and if so, how)?
	Were stakeholders co-authors of the review?
	Please list any other information that you feel is relevant to note about this study’s engagement approach.

### Stage 5: Collating, summarizing, and reporting the results

The research team will decide the best method for analyzing the charted variables once the data have been collected. We anticipate using a qualitative thematic analysis to summarize narratively-collected information pertaining to engagement methods. This will be conducted using a qualitative coding software, and the specific approach will be decided at the time of analysis. Dichotomous or nominal variables will be summarized using descriptive statistics. The results may be presented using figures, concept maps, diagrams, or tables. Regular meetings and consensus discussions will aim to eliminate biases and strive to achieve a mutual interpretation of the review findings. During this stage, scoping reviews may be excluded from the final analyses if they do not provide sufficient detail about the methods used to engage patients and caregivers.

### Stage 6: Consultation

Upon completion of preliminary analyses, we will also consult patients, caregivers, and researchers to gather additional data on methods used to engage patients and caregivers in scoping reviews and help make sense of study findings. We plan to do this by surveying corresponding authors of articles included in our review and patients, caregivers, and researchers recruited through personal (e.g., social media) and professional networks. The survey will re-phrase the review question to directly address respondents and contain items that aim to clarify and/or validate preliminary findings. The survey will also ask respondents to identify scoping reviews of health research that engaged patients and caregiver co-researchers, for consideration for inclusion in the review using the process outlined above. The specific survey questions will be decided once the preliminary analysis has been completed. To improve accessibility, respondents will also be given the option to complete the survey through other modes (e.g., phone call, Zoom) and engage in a follow-up discussion if they wish to build upon their survey responses.

## Discussion

The proposed scoping review will gather existing methods for engaging patients and caregivers in scoping reviews. Due to the limited availability of guiding methods, we expect our review findings to assist researchers in incorporating lived experience perspectives in their scoping reviews. For example, the Preferred Reporting Items for Systematic Review and Meta-Analysis checklist extension for scoping reviews (PRISMA-ScR) contains a checklist for the reporting of essential and optional items for scoping reviews. Though the other elements of Arksey and O’Malley’s framework are represented in PRISMA-ScR, reporting of the goals, activities, and findings of stakeholder consultation are absent [19]. We hope that our study findings support the creation of a set of recommended reporting items related to consultation that could be added to the PRISMA-ScR checklist. Ultimately, we aim to advance and facilitate the inclusion of patient and caregiver perspectives in research so as to improve understanding of their conditions and healthcare experiences, and consequently, the provision of their health care.

We expect our review to show that the methods used to engage patients and caregivers in scoping reviews are heterogeneous, as is their reporting. We also anticipate more frequent consultation at the planning stages of research (i.e., Stage 1: Identifying the research question) and following data analysis, and less often in the actual conduct of the review (e.g., identifying studies, selection, charting, analysis). This is in keeping with other patient engagement research, which has shown that patients are often treated as consultants at key study points rather than partners or co-producers of the research product [23]. Another goal of our review will be to produce recommendations for co-producing scoping reviews with patients from start to finish. We acknowledge the scoping review protocol by Tischerning et al. [24], and systematic rapid review by Manafo et al. [10], which have some shared objectives, but without focus on engagement in the context of a scoping review study design. Our review findings will be disseminated in the form of a peer-reviewed publication, conference presentations, and non-traditional knowledge transitional activities, such as on our research group website (http://www.patientengagementinresearch.ca).

## Supplementary Information

**Additional file 1.** PRISMA-P checklist.

**Additional file 2.** PRISMA-ScR checklist.

**Additional file 3.** GRIPP2 checklist.

## Data Availability

All data generated or analyzed in this study will be included in the published scoping review article. Other resources can be made available upon request.

## Abbreviations

PRISMA-ScR: Preferred reporting items for systematic review and meta-analysis scoping reviews
PRISMA-P: Preferred reporting items for systematic review and meta-analysis protocols
GRIPP2: Guidance for reporting involvement of patients and the public 2

## References

[1] Colquhoun HL, Levac D, O'Brien KK, Straus S, Tricco AC, Perrier L, Kastner M, Moher D (2014). Scoping reviews: time for clarity in definition, methods, and reporting. J Clin Epidemiol.

[2] Anderson S, Allen P, Peckham S, Goodwin N (2008). Asking the right questions: scoping studies in the commissioning of research on the organisation and delivery of health services. Health Res Policy Syst.

[3] Abelson J (2019). Supporting the evaluation of public and patient engagement in health system organizations: results from an implementation research study. Health Expect.

[4] Tricco AC, Lillie E, Zarin W, O’Brien K, Colquhoun H, Kastner M (2016). A scoping review on the conduct and reporting of scoping reviews. BMC Med Res Methodol.

[5] Arksey H, O’Malley L (2005). Scoping studies: towards a methodological framework. Int J Soc Res Methodol Theory Pract.

[6] Levac D, Colquhoun H, O’Brien KK (2010). Scoing studies: advancing the methodology. BMC Implement Sci.

[7] Colquhoun HL, Levac D, O’Brien KK, Straus S, Tricco AC, Perrier L (2014). Scoping reviews: time for clarity in definition, methods, and reporting. J Clin Epidemiol.

[8] Pham MT, Rajić A, Greig JD, Sargeant JM, Papadopoulos A, Mcewen SA (2014). A scoping review of scoping reviews: advancing the approach and enhancing the consistency. Res Synth Methods.

[9] Crockett LK, Shimmin C, Wittmeier KDM, Sibley KM (2019). Engaging patients and the public in Health Research: experiences, perceptions and training needs among Manitoba health researchers. Res Involv Engagem.

[10] Manafò E, Petermann L, Vandall-Walker V, Mason-Lai P (2018). Patient and public engagement in priority setting: a systematic rapid review of the literature. PLoS ONE.

[11] International Association for Public Participation. IAP2 spectrum. CIHR.

[12] Canadian Institutes of Health Research (CIHR). Strategy for patient-oriented research (SPOR) patient engagement framework. CIHR. 2014.

[13] Duffett L (2017). Patient engagement: what partnering with patient in research is all about. Thromb Res.

[14] Vat LE, Finlay T, Jan Schuitmaker-Warnaar T, Fahy N, Robinson P, Boudes M (2020). Evaluating the “return on patient engagement initiatives” in medicines research and development: a literature review. Health Expect.

[15] Oravec N, Arora RC, Bjorklund B, Gregora A, Monnin C, Duhamel TA (2021). Expanding enhanced recovery protocols for cardiac surgery to include the patient voice: a scoping review protocol. Syst Rev.

[16] Oravec N, Arora RC, Bjorklung B (2021). Patient and caregiver preferences for cardiac surgery: a scoping review and consultation workshop. J Thorac Cardiovasc Surg.

[17] Abelson J, Li K, Wilson G (2015). Supporting quality public and patient engagement in health system organizations: development and usability testing of the Patient and Public Engagement Evaluation Tool. Health Expect.

[18] Staniszewska S, Brett J, Simera I, Seers K, Mockford C, Goodlad S (2017). GRIPP2 reporting checklists: tools to improve reporting of patient and public involvement in research. BMJ.

[19] Tricco AC, Lillie E, Zarin W, O’Brien KK, Colquhoun H, Levac D (2018). PRISMA extension for scoping reviews (PRISMA-ScR): checklist and explanation. Ann Intern Med.

[20] Decoster K, Appelmans A, Hill P (2012). A health systems research mapping exercise in 26 low- and middle-income countries: narratives from health systems researchers, policy brokers and policy-makers.

[21] Canadian Institutes of Health Research. Health Research in Canada and You. https://cihr-irsc.gc.ca/e/43753.html [updated 2015-01-12, cited 2022-06-05].

[22] McGowan J, Sampson M, Salzwedel DM, Cogo E, Foerster V, Lefebvre C (2016). PRESS peer review of electronic search strategies: 2015 guideline statement. J Clin Epidemiol.

[23] Domecq JP, Prutsky G, Elraiyah T, Wang Z, Nabhan M, Shippee N (2014). Patient engagement in research: a systematic review. BMC Health Serv Res.

[24] Tscherning SC, Bekker HL, Vedelo TW (2021). How to engage patient partners in health service research: a scoping review protocol. Res Involv Engagem.

