# Supplementary material for: Protocol for a scoping review to map patient engagement in scoping reviews
Source: Res Involv Engagem. 2022 Jun 20;8:27. doi: 10.1186/s40900-022-00361-x (PMC9210720; doi:10.1186/s40900-022-00361-x)
Supplement: Supplementary file 3 — Additional file 3. GRIPP2 checklist. [file 40900_2022_361_MOESM3_ESM.docx]

| Section and topic | Item | Reported on page No |
| --- | --- | --- |
| 1: Aim | Report the aim of PPI in the study | 9 |
| 2: Methods | Provide a clear description of the methods used for PPI in the study, including both positive and negative outcomes | 9 |
| 4: Discussion and conclusions | Outcomes—comment on the extent to which PPI influenced the study overall. Describe positive and negative effects | 9, 24-25 |
| 5: Reflections/critical perspective | Comment critically on the study, reflecting on the things that went well and those that did not, so others can learn from the experience | 9, 24-25 |

Additional file 3. GRIPP2 short form.

PPI = patient and public involvement
